# The Interplay Between the Ubiquitin–Proteasome System and Oxidative Stress: A Future Perspective in Eye Diseases

**DOI:** 10.3390/antiox14121475

**Published:** 2025-12-09

**Authors:** Grazia Raffaella Tundo, Gabriele Antonio Zingale, Irene Pandino, Elisa Peroni, Diego Sbardella, Alessio Bocedi

**Affiliations:** 1 IRCCS-Fondazione Bietti, Via Livenza, 3, 00198 Rome, Italy; grazia.tundo@fondazionebietti.it (G.R.T.); gabriele.zingale@fondazionebietti.it (G.A.Z.); irene.pandino@fondazionebietti.it (I.P.); diego.sbardella@fondazionebietti.it (D.S.); 2 Department of Clinical Sciences and Translational Medicine, University of Tor Vergata, 00133 Rome, Italy; 3 CY Cergy Paris Universitè, CNRS, BioCIS UMR 8076, 95000 Cergy Pointoise, France; elisa.peroni@cyu.fr; 4 Universitè Paris-Saclay, CNRS, BioCIS UMR 8076, 91400 Orsay, France; 5 Department of Chemical Sciences and Technologies, University of Tor Vergata, 00133 Rome, Italy

**Keywords:** proteasome, proteostasis, oxidative stress, antioxidants, eye disease, ophthalmology, neurodegeneration

## Abstract

Redox unbalance, a molecular trait common to neurodegenerative conditions and para-physiological processes like aging, is a critical factor in disease development and in exacerbating progression. The mechanism by which redox imbalance perturbs cellular homeostasis is strongly linked to the activity and function of the ubiquitin–proteasome system (UPS). The UPS, along with autophagy, is the primary intracellular proteolytic system, regulating targeted proteolysis and removing damaged proteins. Consequently, the UPS serves also as the first line of defense for cellular recovery following exposure to redox stressors. Paradoxically, the composition and function of the UPS can also be negatively targeted by redox unbalance through a vicious cycle. The alterations in redox balance and UPS biological mechanisms are involved in the etiopathogenesis of chronic eye disorders. These disorders encompass a diverse repertoire of pathologies affecting the retinal layers (e.g., age-related macular degeneration, diabetic retinopathy) and the optic nerve (e.g., glaucoma). Nowadays, the comprehension of the interplay between proteostasis and oxidative redox status remains pivotal for identifying new therapeutic approaches. Encouragingly, a number of anti-oxidant compounds have been reported to modulate proteasome activity against redox insults in vitro and in vivo. Furthermore, these compounds provide cytoprotective roles in both in vitro and animal models of eye diseases. Therefore, this review highlights recent research on the interplay of the UPS with oxidative stress in physio-pathological conditions, focusing on the onset and progression of ocular diseases, thereby providing new insights into UPS-oxidative stress interaction.

## 1. Introduction

Oxidative stress (OS) and redox unbalance play a pivotal role in the etiology and pathogenesis of numerous human diseases and age-related disorders, including Alzheimer’s, Parkinson’s disease, and cancer [1,2,3]. From a chemical perspective, OS is defined as a redox imbalance resulting from a disruption of the pro-oxidant/anti-oxidant homeostasis, often involving an excess of reactive species, such as reactive oxygen species (ROSs) and reactive nitrogen species (RNSs). While these molecules are naturally occurring metabolic by-products and chemical messengers crucial for physiological processes (such as the effector phase of immune responses), their pathological overabundance causes abnormal oxidation of critical biomolecules. This damage subsequently promotes inflammation, mitochondrial dysfunction, blood-barrier abnormalities, protein oligomerization, and altered protein turnover [2,4]. Therefore, it is immediately clear that the molecular mechanisms by which OS induces a disease state are deeply intertwined with the composition and activity of the cellular pathways responsible for maintaining the proteostasis network and cellular homeostasis. These pathways are the ubiquitin–proteasome system (UPS) and the autophagy–lysosomal pathway (ALP) [5,6]. However, while this pathological scenario is a common feature in eukaryotic cells and tissues, it exhibits unique characteristics and clinical relevance within the visual system. Thus, the eye represents an ideal organ for investigating the redox–proteostasis interplay, since it is exposed to a high oxidative load (e.g., light exposure, elevated oxygen tension, metabolic demand), thereby requiring exceptionally stringent proteostatic control to sustain visual function. Moreover, ocular tissues and fluids are considered as a viable proxy for exploring pathophysiological processes of the deep brain by virtue of their anatomical accessibility. This highlights their critical importance for future research focused on intervening in central nervous system pathways [7].

**Figure 1:**
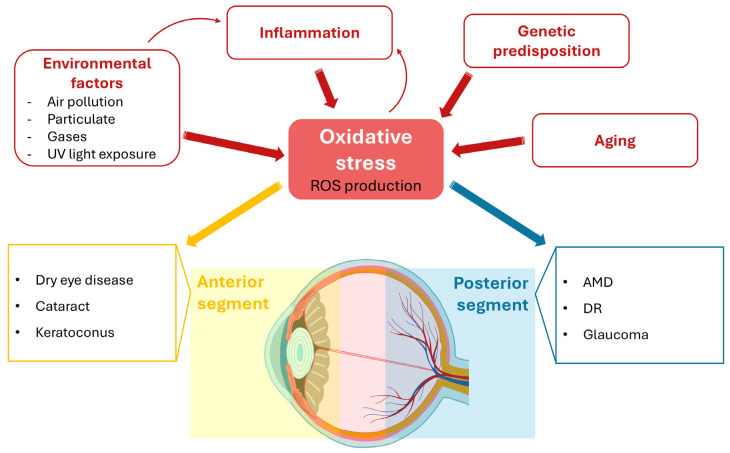
The complex etiology of oxidative stress (OS) in the ocular system. The eye is highly susceptible to OS due to chronic light exposure and high metabolic demands. OS results from an imbalance between reactive oxygen species (ROS) production and anti-oxidant defenses, driven by various factors, including environmental exposure, inflammation, aging, and genetic predisposition. ROS-mediated damage in the anterior segment (cornea, conjunctiva, lens) contributes to conditions such as dry eye disease, cataract, and keratoconus. The posterior segment (retina, optic nerve), which exhibits high metabolic activity and oxygen consumption, is also inherently prone to oxidative damage, leading to various eye diseases, such as age-related macular degeneration (AMD), diabetic retinopathy (DR), and glaucoma.

The anterior segment of the eye (including the conjunctiva, cornea, and lens) is directly exposed to UV radiation and relies on a complex, regulated anti-oxidant defense system (Figure 1) [8,9,10,11]. The breakdown of this delicate balance contributes to the onset of numerous anterior segment pathologies, such as dry eye disease, cataract, and keratoconus [10]. The posterior segment, where the retina and the optic nerve are anatomically located, exhibits exceptionally high metabolic activity and oxygen consumption rates and, therefore, are inherently prone to oxidative damage [12,13]. Furthermore, a variety of factors, including inflammation, aging, genetic predisposition, and environmental pollution, act synergistically to both promote ROS generation and compromise existing anti-oxidant defenses [8]. Hence, it is not surprising that strong preclinical evidence points to redox unbalance and failure in proteolytic systems in the pathogenesis of chronic eye diseases that, all together, represent the prevalent cause of irreversible blindness globally, such as age-related macular degeneration (AMD), glaucoma, and diabetic retinopathy (DR). The involvement of the UPS, whose association with protein accumulation plays a pivotal role in the onset and progression of pathologies affecting both the anterior and posterior segments of the eye, has been recently reviewed elsewhere [14].

In this review, through a literature inspection (see Materials and Methods), we survey existing works concerning the molecular and functional interplay between OS and proteasome modulation in ocular diseases. Despite the recognized pathophysiological relevance of this connection, a substantial gap persists regarding the precise mechanisms through which redox unbalance shapes proteasomal activity in ocular tissues. It is important to note that this gap is also due to the poor availability of in vitro and in vivo models to investigate redox imbalance and proteostasis in ocular tissues.

In fact, most cell-based systems only partially recapitulate the metabolic and age-related complexity of ocular tissues, and oxidative stress is frequently induced by acute chemical stimuli that differ substantially from the chronic, low-grade insults characteristic of human pathology. Likewise, in vivo models reproduce only selected aspects of specific pathologies. Therefore, interspecies differences in retinal architecture, metabolic features as well as UPS composition restrict their translational value. Taken together, to establish a coherent conceptual framework, this review first outlines the structural and functional organization of the UPS and details the direct and indirect modalities through which OS influences proteasome performance. Then, we explore how these processes operate synergistically in the context of the ocular pathologies, including the examination of anti-oxidant compounds known to modulate proteasomal activity. Ultimately, this review seeks to support the rational identification of novel molecular targets and to encourage future investigations aimed at elucidating the intricate crosstalk between redox signaling and proteasomal regulation within the eye.

## 2. Materials and Methods

The literature for proteasome and its implication in eye diseases was inspected, and the most closely related published studies were taken into consideration. Moreover, we inspected topics of the Special Issue. We performed the systematic research on PubMed, Web of Science, and Scopus using the following keywords with no restrictions and in Boolean searching mode: proteasome structure, proteasome function, oxidative stress, protesome, ubiquitin–protesome system, ophthalmology, eye, antioxidants, eye’s disease, and animal model. Articles, editorials, letters, and abstracts not directly related to proteasome and eye diseases were excluded. Figures were digitally illustrated and assembled using Microsoft PowerPoint 2024 for layout and composition.

## 3. Ubiquitin–Proteasome System: An Overview

The UPS is the main pathway controlling the turnover of intracellular proteins, thus playing a key role in several phase of cell life, spanning from regulation of cell proliferation and death, DNA repair, inflammation, stressful adaptation, and the morphogenesis of neuronal networks [6,15]. The endpoint of the UPS is the 26S proteasome, a multi-catalytic complex which degrades protein substrates covalently conjugated with ubiquitin polymers [15,16,17]. Ubiquitin conjugation to targeted substrate proceeds through a finely tuned cascade involving the coordinated and ordered action of three enzymes: E1, E2, and E3. The process begins when E1 activates ubiquitin in an ATP-dependent manner, generating a high-energy thiol ester intermediate. In the second step, activated ubiquitin is transferred to an E2 enzyme, creating a second high-energy thiol ester intermediate. Finally, ubiquitin is ligated to a target substrate by a specific ubiquitin ligase E3 which serves as a main control of substrate specificity [16,18]. Different ubiquitin moieties can then be conjugated to the first molecule through various chain types, referred to as M1, K6, K11, K29 K48, and K63 linkages, among others. In this nomenclature, the number identifies the lysine residue (except for M1) involved in the isopeptide bond with the C-terminus of the next ubiquitin moiety along the chain.

Therefore, according to the most characterized UPS degradation feature, proteins covalently tagged with at least four ubiquitin molecules (polyubiquitin chains) joined specifically via a K48 linkage are targeted for degradation by the 26S proteasome [19,20]. Remarkably, it has been recently proposed that alternative signals should address substrates to degradation, such as mono-ubiquitination or specific structural elements. Anyway, the biological relevance of these alternative cues is unknown [21,22,23].

In its canonical form, the 26S is a holocomplex assembled from two main components: the 20S catalytic core and the 19S regulatory particle. The 20S particle is a symmetrical, barrel-shaped structure formed by two outer α-rings and two inner β-rings (i.e., α1-7, β1-7, β1-7, α1-7).

These rings surround a central channel that harbors three major catalytic activities: (i) Chymotrypsin-like (ChT-L): encoded by the β5 subunits, this activity typically cuts peptide bonds after amino acids with large hydrophobic side chains; (ii) Trypsin-like (T-L): encoded by the β2 subunits, this activity preferentially cleaves peptide bonds after basic (positively charged) amino acids at the P1 position (e.g., Lysine (K) or Arginine (R)); and (iii) Caspase-like (C-L) or Peptidylglutamyl-peptide hydrolyzing (PGPH): encoded by the β1 subunits, this activity shows a preference for cleaving peptide bonds after amino acids with acidic side chains, such as Glutamate (E) or Aspartate (D), at the P1 position [24,25].

The 19S, which caps the outer 20S α-rings and consists of two different sub-complexes at the base, directly interacts with the 20S and the external lid, which, instead, carries out the ATP-dependent recognition and unfolding of the polyubiquitinated substrate and its translocation into the 20S central catalytic channel [26,27].

The crucial roles carried out by the proteasome in cell life, demands a tight regulation of its activity and composition at several levels, such as transcriptional regulation, post-synthetic modifications, interaction with a number of regulatory proteins, and the incorporation of alternative subunits in nascent particles [28,29,30]. Concerning the last point, tissue-specific 20S proteasome particles have been identified, so that, within a single cell, canonical and non-canonical forms of proteasome coexist. Each particle is supposed to play distinct biological roles [31]. In this regard, the immunoproteasome is the most characterized inducible form of proteasome expressed in immune cells and in cells, exposed to inflammatory cytokines (i.e., IFN-γ). In this complex, the three catalytic subunits (i.e., LMP2, MECL-1, LMP7) are replaced by their inducible counterparts β1i, β2i, and β5i [32,33]. Moreover, the immunoproteasome is believed to be preferentially associated with a regulatory particle with distinguished structural and functional properties called proteasome activator 28 (PA28) [34,35,36,37]. PA28 is now recognized to be expressed as an heptameric heterodimer of α and β subunits (PA28αβ) or as a heptameric homodimer of γ-subunits (PA28γ) [34]. Besides its pivotal role in MHC class I antigen processing and immune system modulation, a biological function probably carried out by both immunoproteasomes sub-types (e.g., PA28αβ and PA28γ) show, at least in vitro, optimized activity for the degradation of oxidized and intrinsically disordered proteins.

In fact, it has been proposed that oxidized protein turnover is not related to ATP/ubiquitin-dependent degradation and with 26S, even though some controversies still exist [38,39,40,41]. These controversies largely stem from an unresolved debate regarding the biological role of the free 20S proteasome and its functional distinctiveness from the 26S complex. While the 26S has historically received far greater experimental attention, recent biochemical and structural studies indicate that the 20S can exist as an independent and dynamically regulated entity with substrate preferences that differ substantially from those of the 26S holoenzyme. In this context, the degradation of oxidized proteins has become a useful model system to distinguish ubiquitin-dependent from ubiquitin-independent pathways, as several lines of evidence support direct 20S recognition of oxidation-induced conformational changes [42,43]. Specifically, oxidation leads to chemical modifications that induce conformational changes in proteins. These changes expose hydrophobic patches on the protein surface that are subsequently recognized by the free 20S. This recognition is followed by the channeling of the substrate into the 20S catalytic chamber for degradation [42,43,44].

Conversely, other studies argue that specific oxidized substrates still require ubiquitin tagging or 19S assistance for efficient turnover. These divergent observations likely reflect differences in substrate identity, oxidative modifications, and experimental systems, highlighting the need for more physiologically relevant models to resolve the distinct contributions of 20S versus 26S in oxidative proteostasis. Under this stressful condition, it was hypothesized that the recruitment of specific proteasome interacting proteins (PIPs) such as Ecm29 or DJ-1 facilitates 26S dissociation, increasing the abundance of the free 20S and its ubiquitin-independent proteolytic activity [43,45]. It is worth pointing out that, in addition to oxidized/unfolded proteins, very recently, the free 20S has been proposed to cleave also mono-ubiquitylated proteins releasing branched protein: ubiquitin fragments [20,46]. Whether this completely new proteolytic activity has major biological roles in vivo still demands the development of tools and experimental models which unequivocally support this possibility. However, protein models on which studies were run, including cyclin B1 protein and tauK18 (i.e., a domain of tau protein/MAPT gene covering the four-repeat region of the protein) are concordant with the general biochemical mechanisms for the substrate specificity of this particle, and for the contribution of oxidative stress to its activity [20].

Besides promoting protein unfolding, persistent OS also causes proteasome impairment, by chemical modification of the catalytic subunits, transient inactivation of E1, E2, and E3 enzymes and 26S disassembly.

**Figure 2:**
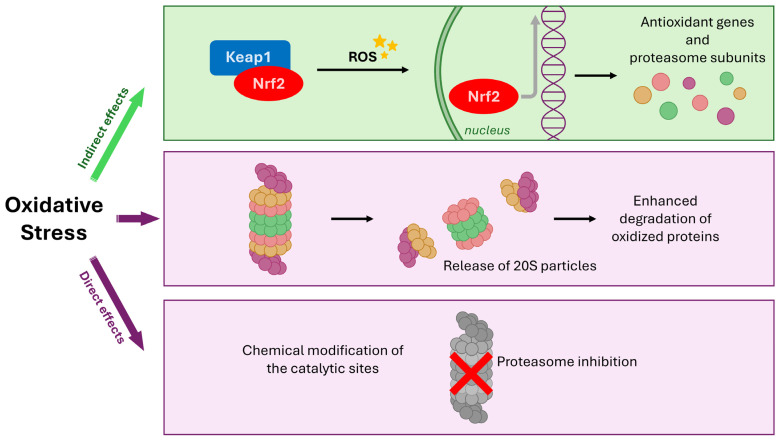
Regulation of the proteasome by oxidative stress (OS) through direct damage and indirect nuclear factor erythroid 2-related factor 2 (Nrf2) signaling. OS directly impacts the proteasome via two main effects: inhibition due to chemical modification of catalytic sites, and potential release of proteasome particles leading to enhanced degradation of oxidized proteins. Concurrently, OS acts indirectly by causing the oxidation of Kelch-like ECH-associated protein 1 (Keap1), liberating Nrf2. Nuclear translocation of Nrf2 initiates the transcription of stress-responsive genes, including those encoding proteasome components, in an effort to upregulate the clearance system and compensate for OS-induced damage and overload.

The OS-related dysfunction of the proteasome establishes a detrimental self-perpetuating cycle in which decreased clearance of oxidized proteins further amplifies the intracellular stress burden. In the attempt to counteract these direct effects, ROS accumulation triggers a complex cellular stress response that can indirectly stimulate proteasome activity. The Nuclear factor erythroid 2-related factor 2 (Nrf2) is the main transcriptional factor which drives cellular response to OS. Under physiological conditions, Nrf2, in complex with its repressor Keap1, remains in the cytosol. Oxidation of Keap1 induces the translocation of Nrf2 to the nucleus, where it induces the transcription of anti-oxidant and detoxification genes. Notably, Nrf2 also promotes the expression of certain gene-encoding subunits of the 20S and 19S proteasome, representing a feedback loop where oxidative stress indirectly upregulates proteasome components in an attempt to compensate for its damage and overload (Figure 2) [47,48,49].

Consequently, in light of the aforementioned implication of OS in the etiology and pathogenesis of a spectrum of human diseases (please see also the Box 1), the elucidation of the regulatory mechanisms governing the proteasome-mediated degradation of oxidized proteins offers the potential to yield novel therapeutic and prognostic paradigms for these conditions.

Box 1 **A look at the risk factors driving oxidative stress in human diseases.**

      OS is one of the most studied phenomena standing at the intersection of cell biology, cell physiology, and general biochemistry. The first consequence of electron transfer reactions in living systems is the transient generation of free radicals. Very recently, some authors pointed out that OS is a “double-edged sword” for living systems. On one hand, OS in the form of “oxidative eustress” contributes to physiological signaling and is sustained by controlled concentrations of ROS; on the other hand, “oxidative (di)stress” reflects excessive ROS levels that mediate oxidative damage to biomolecules [50]. As previously reported in the Introduction (Section 1), OS is defined as a redox imbalance between pro-oxidant and anti-oxidant systems that perturbs cellular homeostasis [51,52]. Through this imbalance, OS promotes disease onset and progression by inducing lipid peroxidation, structural and functional damage to biological macromolecules, and sustained inflammatory responses. OS also plays a fundamental role in impairing proteostasis, as ROSs and RNSs directly oxidize proteins, lipids, and nucleic acids, triggering misfolding, aggregation, and excessive workload for the proteolytic systems (UPS and autophagy). The risk factors for OS can be categorized into environmental/lifestyle factors (exogenous sources) and internal physiological conditions (endogenous sources). Exogenous sources include the following: unhealthy diets, sedentary lifestyle, smoking, alcohol consumption, stress, pollution and radiation [51,52]. Given the prominence of this topic, several reviews have addressed the risk factors underlying OS and their association with various disorders. Here, we briefly outline the most frequently cited examples, without aiming to be exhaustive, and direct the reader to more specialized reviews for a deeper discussion. Exposure to air pollutants, heavy metals, xenobiotics, and toxins increases OS by generating free radicals and impairing anti-oxidant defenses. Cigarette smoke contains high concentrations of reactive chemicals that directly elevate ROS levels and trigger inflammatory pathways. Diets rich in calories, saturated fats, and processed foods—as well as excessive alcohol intake—enhance mitochondrial ROS generation. Prolonged exposure to UV or ionizing radiation stimulates ROS formation and promotes DNA and cellular injury. Endogenous factors playing a relevant role are chronic inflammation, aging, genetic predispositions, pre-existing metabolic conditions such as obesity and diabetes, and sleep disorders [51]. As matter of fact, chronic activation of immune cells leads to ROS production as part of host defense mechanisms; however, when unrestrained, this results in persistent OS. Metabolic disorders are also characterized by amplified OS and low-grade chronic inflammation. Additionally, hyperglycemia in diabetes causes intracellular glucose overload and mitochondrial electron leakage, boosting free radical production. Finally, aging naturally leads to the gradual accumulation of oxidative damage over time, further sensitizing tissues to OS-related dysfunction [51,52]. During aging, the decline of proteasome activity, accumulation of damaged mitochondria, and increased cellular senescence further exacerbate OS, establishing a reciprocal reinforcement between oxidative imbalance and impaired proteostasis (see the main text for details).

## 4. The Interplay Between Proteasome and Oxidative Stress in Ocular Diseases

**Figure 3:**
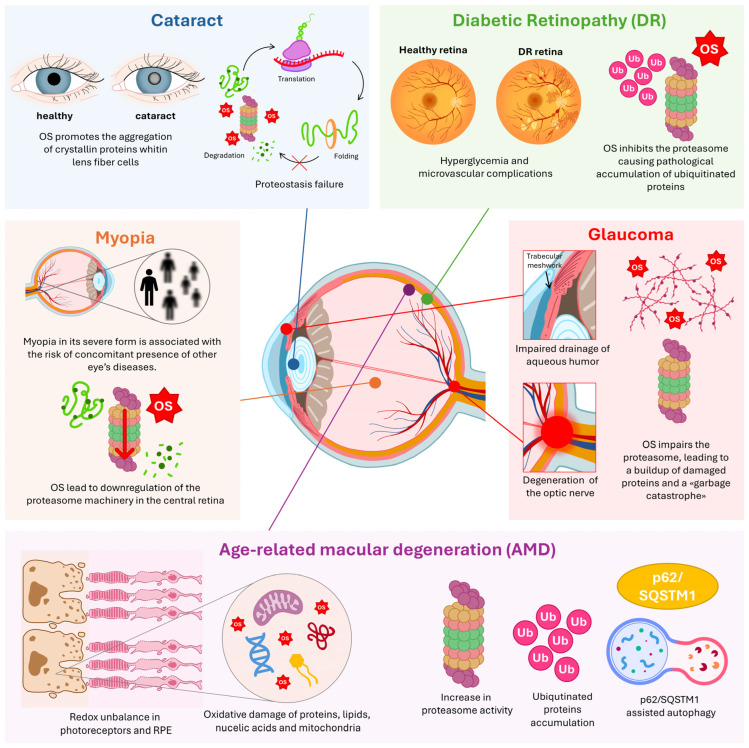
Molecular landscape of ocular diseases and clinical outcomes. Panoramic representation of all cited eye diseases, their associated clinical outcomes, and the underlying molecular processes. Key emphasis is placed on oxidative stress (OS) mechanisms involving the proteasome and the Ubiquitin–Proteasome System (UPS), underscoring their significance in ocular health and disease. RPE: Retinal Pigment Epithelium; p62/SQSTM1: Sequestosome-1; Ub: Ubiquitin.

The most vulnerable and critical cellular system in the context of retinal pathologies is the Retinal Pigment Epithelium (RPE). This essential monolayer of cells forms the outer blood–retinal barrier between the photoreceptors and the Bruch’s membrane, making it the metabolic hub of the outer retina [53,54] (Figure 3). Given its location and functions, which include continuous high metabolic activity, exposure to light, and the daily phagocytosis of photoreceptor outer segments, the RPE is uniquely susceptible to OS. In fact, chronic OS-induced damage, particularly to mitochondria, eventually leads to the breakdown [55] of cellular quality control, or proteostasis perturbation in RPE cell [56]. Given the marked susceptibility of ocular tissues to oxidative stress, it is not surprising, as mentioned in the preceding sections, that redox imbalance is implicated in the initiation and progression of multiple ocular diseases, and that such dysregulation is closely intertwined with disturbances in cellular proteostasis. The following sections summarize the current body of evidence describing the interplay between these two systems in the context of age-related macular degeneration, cataract, diabetic retinopathy, glaucoma, and myopia.

### 4.1. Age-Related Macular Degeneration

It is well known that chronic OS is a major factor contributing to retinopathies, most notably Age-related Macular Degeneration (AMD), the leading cause of irreversible blindness globally, as well as Retinitis Pigmentosa and Stargardt disease (SD) [53,54,57]. Accordingly, OS and alteration of proteostasis are hallmarks of AMD, witnessed by the intracellular accumulation of lipofuscin in lysosomes and the extracellular deposition of drusen [58,59]. To counteract continuous oxidative molecular damage, the RPE and retinal endothelial cells, with which RPE has intense crosstalk, rely on the UPS and ALPs for which complex interplay in maintaining RPE homeostasis and integrity has been reported (Figure 3) [58,60].

In early-intermediate AMD, corresponding to stage 3 of Minnesota Grading System, evidence suggests that RPE initially mounts a vigorous defense against OS. In fact, the expression of proteins that fight this stress (including those of the UPS and ALP), is significantly increased [57,58,59].

However, this tentative defense ultimately fails: accumulation of ubiquitinated proteins within drusen beneath the macula of late-stage AMD donors suggests a state of proteasome saturation or inhibition [61].

From molecular point of view, the previously discussed interplay between UPS and ALP is structurally and functionally coordinated by proteins such as the chaperone Hsp70 and the cargo receptor SQSTM1/p62 [62]. SQSTM1/p62 contains ubiquitin- and LC3-binding domains, allowing it to selectively target ubiquitinated aggregates to either the proteasome or the autophagosome, thereby coordinating the two major catabolic pathways [62]. When proteasome is inhibited in RPE, ubiquitin conjugates and Hsp70 accumulate perinuclearly, coinciding with an increase in LAMP-2-positive lysosomes [63,64]. This inhibition signals the induction of p62-assisted autophagy to clear the aggregated cargo [65].

The UPS and ALP mutual regulation is further highlighted by the evidence that mTORC1 (Mammalian Target of Rapamycin Complex 1) inhibition, which typically promotes autophagy, has been shown to simultaneously increase UPS protein degradation [66].

Pharmacological inhibition of proteasome with MG132 was shown to stabilize lysosomes, to induce autophagy, and ultimately to protect RPE cells from oxidative damage in vitro, suggesting that ALP can compensate for UPS deficit [67]. While direct proteasome inhibition therapies are unlikely to be clinically feasible due to the proteolytic system’s critical importance for retinal ganglion cell (RGC) survival, these findings validate the concept that targeted regulation of specific ubiquitylation cascades may offer new therapeutic avenues. It should be noted that in some cases, the literature reports divergent outcomes regarding proteasome inhibition under oxidative stress conditions. In some experimental systems, partial UPS suppression appears cytoprotective by activating compensatory autophagy, whereas in others it exacerbates proteotoxic stress. These discrepancies are likely context-dependent and may reflect differences in cell type, degree of proteasome inhibition, baseline redox burden, or differential engagement of ALP-mediated clearance pathways.

The coordinated response between UPS and ALP is further underscored by the EI24 (etoposide-induced protein 2.4 homolog) ubiquitin ligase, which has been proposed to link the two systems by promoting the degradation of specific RING E3 ligases within the autophagic process [55]. Critically, bioinformatics analysis substantiates this paradigm of the integrated stress response, and is supported by enrichment in pathways and processes governing the coordinated function of UPS and ALP [68].

The critical link between OS and proteostasis failure in AMD is supported also by mechanisms of transcriptional control. The detoxification transcription factors NRF-2, which regulates the transcription of proteasome subunits (see Section 2), and peroxisome proliferator-activated receptor gamma coactivator-1 alpha (PGC-1α), which regulates autophagy genes, are both essential for RPE health. The NRF-2/PGC-1α double knockout (dKO) mouse model develops an AMD-like phenotype, exhibiting RPE degeneration, mitochondrial dysfunction, and increased level of markers of both OS (i.e., 4-Hydroxynonenal) and proteostasis failure (i.e., SQSTM1/p62, Beclin-1, LC3B) [68,69].

Ultimately, chronic proteostasis failure is believed to intersect with inflammation in RPE. High OS can trigger the assembly of the NLRP3 inflammasome complex [56]. The resultant lysosomal destabilization, caused by accumulated lipofuscin-derived danger signals (DAMPs), activates caspase-1, leading to the release of pro-inflammatory cytokines (IL-1β and IL-18) that drive chronic inflammation and pyroptotic RPE cell death, which is a central pathological process in advanced AMD [70]. Another hallmark of age-associated AMD is the advanced glycation end product (AGE) deposition in drusen and in Bruch’s membrane of the eye. A transcriptomic study revealed that, in RPE cells, in response to AGEs, gene involved in inflammation and proteasome degradation are induced [71]. Moreover, in RPE, monocyte chemoattractant protein-1 (MCP-1) level, whose expression is regulated by NFkB activation, decreases after the treatment with proteasome inhibitor MG-132 [72]. In platelet-activating factor (PAF)-stimulated ARPE-19 cells, the depletion of PKC-α was prevented by administration of proteasome inhibitors lactacystin and MG-132, suggesting that the PAF-induced downregulation of PKC occurs through the proteasomal pathway [73]. Taken together, these findings highlight an unresolved controversy: UPS activity can either amplify inflammatory signaling or attenuate it, depending on the specific substrates involved and the prevailing redox environment. Such bidirectional effects underscore the need to better define selective ubiquitylation events in the RPE, particularly under chronic oxidative conditions.

Complementary to this, Factor H (a key component of the complement cascade) was found to directly interact with UPS components in RPE cells in a complement-independent manner, providing a critical molecular link between the immune and proteolytic systems [66].

The UPS’s contribution to retinal OS and pathology is clearly demonstrated by research focusing on non-canonical proteasome particles, particularly the immunoproteasome.

This particle is distinguished by its inducible catalytic subunits: LMP2 (β1i), MECL−1 (β2i), and LMP7 (β5i) (see Section 3). The mechanistic role of immunoproteasome in retinopathy is supported by some preclinical evidence. Studies investigating cultured RPE cells from KO mice deficient in one (lmp7^−/−^) or two (lmp7^−/−^ + mecl−1^−/−^) subunits revealed that OS exposure promotes effective immunoproteasome upregulation only when all catalytic subunits are present. This finding suggests that a specific and concerted mechanism is required for their effective incorporation into newly synthesized particles, which has represented a finding of broader biological significance [74].

Moreover, the immunoproteasome was found to significantly contribute to stress-related diseases like Angiotensin II (Ang II)-induced retinopathy (a model for hypertensive retinopathy). Research in Angiotensin II (Ang II)-infused mice and human hypertensive retinopathy patients showed a significant increase in the immunoproteasome subunit LMP10 (β2i subunit) expression and its trypsin-like activity in both the retina of enrolled animals and serum of human subjects [75].

In the Ang II mouse model, high LMP10 exacerbated retinal damage, including increased central retinal thickness, vascular permeability, inflammation, and oxidative stress (ROS). Importantly, genetically eliminating LMP10 (LMP10 KO mice) significantly reduced these effects, while artificially increasing LMP10 (rAAV2-LMP10 injection) worsened them. Mechanistically, Ang II-induced LMP10 upregulates a destructive signaling cascade (PTEN degradation followed by AKT/IKK activation and NF-κB activation), which drives the retinopathy. Blocking a key step in this pathway with the IKKβ inhibitor IMD-0354 effectively reduced the vascular, oxidative, and inflammatory damage [75,76].

Building on data that LMP10/β2i contributes to Ang-II induced retinopathy, the catalytic β5i subunit was found to be upregulated in mice and patients as well. Again, deletion of β5i protected against retinopathy, while its overexpression worsened the disease.

Mechanistically, β5i was found to promote the degradation of ATRAP (AT 1 Receptor−Associated Protein), thereby activating AT1R (Angiotensin II Type 1 Receptor)-mediated signals, which in turn activatess receptor signaling, driving the retinopathy [77].

Moreover, while the concentrations of the PA700 regulatory complex and the LMP7 subunit were shown to remain relatively stable in old rats, the age-associated loss of proteasome activity appeared to be driven by a consistent reduction in the overall expression of 20S catalytic subunits [78].

UPS relevance in maintaining retinal homeostasis is further highlighted by its role in para-physiological processes such as aging. Knockout of the essential ubiquitin protein ligase E3D (UBE3D) led to the progressive development of AMD-like pathology, including granular deposits and photoreceptor deterioration, by failing to properly modulate the metabolism of melanin, melanosomes, and lipofuscin through its interaction with the autophagy and lysosomal systems [79].

### 4.2. Cataract

OS plays a central role in cataract development by promoting the aggregation of crystallin proteins within lens fiber cells, thereby compromising the lens’s transparency and light refraction properties (Figure 3). This pathological process is exacerbated by OS-induced damage, which limits the ability of the lens’s post-mitotic fiber cells to repair or replace damaged crystallins. Furthermore, cataractogenesis is characterized by the failure of programmed organelle clearance, a process that normally occurs as fiber cells mature and is typically regulated by the ALP in several cell lineages [80,81,82,83]. Consequently, proteostasis failure is a key mechanism also underlying cataract formation [81,84].

A study from two decades ago showed the interplay between crystallin aggregates and UPS in familial amyloidotic polyneuropathy, and also UPS impairment linked to unfolded⁄aggregated proteins inside cells [85].

The use of the lens of a particular animal model such as ground squirrels contributed to elucidating that UPS plays a role in minimizing the aggregation of the lens protein αA-crystallin during a temperature-controlled experiments in the phase of rewarming. The E3 ubiquitin ligase, RNF114, appears a key mechanism mediating the turnover and homeostasis of lens proteins. A deliverable RNF114 complex reduced lens opacity in rats with cold-induced cataracts and zebrafish with oxidative stress-related cataracts [84]. A cytoprotective effect in lens cells that are implicated in pathological conditions associated with the cataract was observed after the overexpression of the proteasome catalytic β5 subunit (PSMB5) (see Section 3) and the subsequent coordinated induction of the additional catalytic subunits β1 and β2, improving proteasome activity. Nonetheless, this complex is downregulated during the process of aging of the lens.

In this view, boosting proteasome activity could be a therapeutic approach to block cataract formation and progression, while at the same time increasing cell resistance to oxidative stress effects [86]. Additional scientific evidence linking OS, proteostasis, and cataracts comes from diabetic cataracts. This condition is fueled by redox unbalance triggered by high blood glucose with a critical contribution of HIF−1α (Hypoxia-Inducible Factor-1 alpha) transcriptional signal [80].

A study on SRA01/04 human lens epithelial cells investigated how high glucose enhances HIF-1α protection via SUMOylation. High-glucose treatment induced SUMO (Small Ubiquitin-like Modifier) isoforms and the SUMO E3 ligases Cbx4 (Chromobox Homolog 4) and PIASy (Protein Inhibitor of Activated STAT Y), thereby enhancing HIF-1α SUMOylation. Critically, the proteasome inhibitor MG132 protected HIF-1α’s stability and transcriptional activity in these cells. These SUMOylation and UPS processes collectively inhibit apoptosis and protect against lens opacification [80,87,88].

Beyond HIF-1α stabilization, the UPS also controls the fate of other cytoprotective molecules. Lens epithelial cells are protected from OS by the activity of the enzyme Glutathione S-Transferase P1 (GSTP1), whose overexpression was found to attenuate the frequence of apoptotic cells after H_2_O_2_ exposure. However, cataractogenesis is associated with enhanced degradation of this protective enzyme, and a novel association linking the E3 ubiquitin ligase Parkin and GSTP1 ubiquitylation was proposed [82].

The complexity of UPS organization and biological properties is further underscored by the evidence showing that not all interventions that aim at rescuing or improving its functionality are successful. For instance, the overexpression of the proteasome activator PA28αβ (see Section 3), despite its presumed protective role against OS, had no effect on either age-related or H_2_O_2_-induced cataracts in mouse models, concluding that PA28αβ does not protect mice from developing this pathological condition. This lack of protection may reflect several biological factors. In particular, PA28αβ appears to share anti-aggregation properties with α-crystallins, suggesting that its overexpression may not provide an additive benefit in lens fiber cells. Moreover, cataractogenesis triggered by physiological aging in vivo and by H_2_O_2_ exposure ex vivo follows distinct structural and temporal trajectories, indicating that model-specific mechanisms or compensatory responses may mask potential PA28αβ-dependent effects [81,82,83].

### 4.3. Diabetic Retinopathy

Diabetic retinopathy (DR), a microvascular complication of diabetes and a major cause of vision loss, is fundamentally driven by hyperglycemia and resulting high levels of redox unbalance [89].

DR pathology, which progresses through different clinical phases often referred to as non-proliferative and proliferative stages, involves the progressive alteration of vessel hemodynamic and microarchitecture, pericyte loss and endothelial cell proliferation (neo-angiogenesis).

Central to these pathological processes is the role of Muller glia (e.g., the resident microglia of the retina) cell activation, and the release of pro-inflammatory factors [89].

Hyperglycemia and ROS production are intersected through different molecular mechanisms.

When glucose is excessively abundant, it overwhelms the normal energy production pathway (glycolysis and mitochondrial respiration). The excess of glucose is shunted into “collateral” metabolic pathways, including the polyol pathway, the hexosamine pathway, and the production of AGEs, which all contribute to ROS overproduction.

The UPS is centrally involved in diabetes progression: decreased proteasome function in hyperglycemia contributes to ER (Endoplasmic Reticulum) stress, dysfunction, and apoptosis, initially observed in pancreatic β-cells [90].

In the retina, OS and, in general, chronic stress directly impair proteasome function. The regulatory PA700 complex makes the 26S susceptible to OS, impairing its ATP-dependent proteolysis. Although the 20S proteasome is more resistant, its degradation activity and substrate specificity are altered by chronic hypoxia [85].

During DR, increased ROS and RNS, such as peroxynitrite, lead to inactivation of the 20S proteasome and a pathological accumulation of ubiquitinated proteins [90]. These conditions disrupt the degradation of key regulatory proteins (Figure 3). In diabetic mouse models, retinal OS increases the expression of the stress-response protein REDD1 (Regulated in Development and DNA Damage Response 1), which is linked to visual deficits. REDD1 undergoes OS-induced oxidation of intramolecular cysteines, forming a disulfide bond that enhances its accumulation by suppressing its degradation via chaperone-mediated autophagy. Although REDD1 is normally ubiquitinated and targeted to the 26S by E3 ligases, hyperglycemic/oxidative conditions act additively with proteasomal inhibition to suppress its degradation [91].

Further evidence highlights the importance of differential UPS activity in the microvasculature. Retinal endothelial cells exhibit a significantly higher proteasome peptidase activity compared to pericytes, a factor that contributes to the heightened sensitivity of pericytes to high glucose-mediated OS. High glucose directly influences cellular proteasome function by increasing the levels of total ubiquitin-conjugated proteins and upregulating the PA28-α/-β and PA28-β/-γ proteasome regulatory proteins in the retina of diabetic mice [92]. Research using transgenic mice to overexpress PA28 genes in the pericyte compartment is ongoing to clarify the specific role of elevated PA28 proteins in DR development [93].

In this framework, specific research on Muller glia and primary retinal explants has been conducted by our laboratory over the last years. Whilst Muller glia cells are recognized to acquire a pro-inflammatory phenotype after exposure to high glucose concentrations (e.g., 25 mmol/L) for 24–48h, our studies highlighted that as low as 15 min of glucose stimulation triggers a signaling cascade regulated by a Ca^2+^ calmodulin dependent kinase and involving the phosphorylation of the serine 120 of the Rpt6 subunit of the 19S regulatory particle. Phosphorylation of this subunit was associated with increased proteolytic activation and clearance of IkBα, thereby releasing the p65–p50 heterodimer and transcription of NF-kB target genes. A molecular characterization of the pathway uncovered that IL-8, Il-1β, and MCP1 were among the cytokines/chemokines showing the highest extent of transcriptional upregulation in response to glucose stimulation [94,95].

### 4.4. Glaucoma

Glaucoma is a chronic optic neuropathy characterized by progressive death of RGCs and degeneration of the optic nerve. Glaucoma progression follows a pattern common to brain neurodegeneration, in particular, Alzheimer’s disease [7].

Accordingly, multi-faceted alterations of redox unbalance have been widely reported in cell-based experimental models and murine models of the disease for both the posterior and anterior chamber of eye tissues [7].

However, molecular evidence supporting a direct crosstalk between the UPS and redox unbalance has been reported in a limited repertoire of published papers.

To introduce the main findings, it is worth recalling that, regarding the anterior chamber of the eye, a major risk factor of glaucoma, and the only one modifiable by therapeutic intervention, is increased intraocular pressure (IOP).

IOP is primary regulated through production and outflow of the aqueous humor, a fluid which flows through the anterior chamber of the eye, providing nutritional support to the cell types lining the tissues and conferring shape to the eye globe.

In glaucoma, the drainage of aqueous humor and the outflow pathway constituted by trabecular meshwork (TM) and Schlemm’s canal are compromised (Figure 3). TM deposition and homeostasis are primary regulated by the anabolic and catabolic activity of Trabecular Meshwork Cells (TMCs), which are endothelial-like phagocytic cells.

Chronic OS may be one of the contributors to this outflow pathway malfunction and consequently, this leads to an increase in IOP.

After the exposition of human TM cells to the effect of chronic OS, an evident decline in proteasome function was observed, either from inactivation of the proteasome or from a decrease in the number of proteasome units in TM cells [46,96].

In this regard, a progressive malfunction of TM/Schlemm’s canal outflow pathway in primary open-angle glaucoma as a consequence of a “garbage catastrophe” system was proposed. According to this model, damaged and aggregating proteins are the result of an increase in OS and a functional decline in the cellular proteolytic machinery that eliminates misfolded and damaged proteins [97].

More recently, ocular hypertension-related molecular risk factors for glaucoma were investigated by shot-gun proteomics and alterations in the ocular hypertensive human retina. Through this approach, >2000 retinal proteins were identified and quantified. Among upregulated proteins and biological clusters, the authors identified heat shock proteins, ubiquitin–proteasome pathway components, anti-oxidants, and DNA repair enzymes, while the downregulated proteins and biological clusters included mitochondrial oxidative phosphorylation in the ocular hypertensive retina, with respect to the normotensive controls [46,98,99] (Figure 3).

The role of p38MAPK after treatment of human TMCs by the ROS generator tert-butyl hydroperoxide (tBHP) gave interesting results for chymotrypsin-like proteasome inactivation for 1 h of exposition of human trabecular meshwork cells to tBHP and activation for 2 h exposition. In both conditions, the pretreatment with the inhibitor of MAP kinase SB203580 was made with respect to the control, without the MAPK inhibitor [46,100].

### 4.5. Myopia

Myopia in its severe form is associated with the risk of the concomitant presence of other eye diseases. In the literature, the evidence of oxidative stress participation in the development of myopia is reported [101]. Recently, a proteomic study shared some results about the proteome changes in retina. The most significant changes were in the far-periphery retina after myopia induction, followed by the central retina. Proteasome pathway was significantly downregulated in differentially expressed proteins in the central retina [102] (Figure 3).

## 5. Modulation of Proteasome Function by Anti-Oxidants in Eye Diseases

The evolving clinical management of different eye diseases often connected to the aging process incorporates multidisciplinary therapeutic strategies, including senolytics, senomorphics, small molecules, and drug-encapsulating nanoparticles, all aiming to restore cellular function and delay aging [103].

A unifying, central focus in these approaches is the critical and intricate relationship between the management of redox homeostasis and adequate preservation or rescue of the UPS [103].

Thus, with caveats already having been discussed in the previous sections regarding the concrete applicability of proteasome inhibition for the cure of retinal diseases, compelling evidence for a direct therapeutic potential of proteasome modulation comes from studies utilizing specific inhibitors of proteasome catalytic activity. Mild inhibition of the proteasome can surprisingly strengthen cell’s ability to survive stress. This beneficial effect is an example of hormesis, suggesting that low-level stress activates protective cellular responses to guard against future, more severe exposures.

Accordingly, low-dose treatment with proteasome inhibitors is anticipated to trigger the compensatory upregulation of cytoprotective mechanisms. This includes the induction of proteasome subunits to assemble new particles [6,104].

This protective mechanism was further validated by the clinical inhibitor Bortezomib, which was found to attenuate retinal ischemia–reperfusion injury by blocking proteasome activity, thereby linking proteasome inhibition directly to anti-inflammatory and anti-oxidative effects [105].

As matter of fact, in accordance with the intense crosstalk between the UPS/ALP and inflammation, the effects of proteasome inhibitors were found in all these pathways and processes.

In ARPE-19, an RPE cell strain, pretreatment with low-dose proteasome inhibitors like MG-132 and clasto-lactacystin-lactone effectively reduce oxidative damage caused by menadione or 4-HNE. Interestingly, the protective mechanism of MG-132 involves PPARα-dependent pathways, while that of clasto-lactacystin-lactone was PPARα-independent [106].

Crucially, in diabetic rats, MG-132-mediated proteasome inhibition also alleviated retinal vascular injury, which in turn promoted the upregulation of the Nrf2 pathway (see Section 3), as evidenced by increased levels of Nrf2, NADPH-quinone oxidoreductase (NQO1), and Heme Oxygenase (HO)-1 [107].

Non-lethal doses of proteasome inhibitors (such as clasto-lactacystin-lactone or epoxomicin) effectively induced the critical pro-survival mechanism of autophagic flux in ARPE-19 cells, by suppressing the PI3K-Akt-mTOR signaling cascade [108,109].

Similarly, the potential effect in AMD therapy has been proposed for the polyphenol resveratrol, known to induce autophagy, which induces, analogously to a proteasome inhibitor, pro-survival mechanisms and does not show additive effects with MG-132 [110].

Additionally, the use of anti-oxidants N-acetyl-L-cysteine (NAC) and (2R,4R)-4-Aminopyrrolidine-2,4-dicarboxylic acid (APDC) in MG-132 or epoxomicin pre-treated RPE cells reduces the cytotoxicity induced by proteasome inhibitor treatment [108,111].

Contextually, a number of anti-oxidants of various types, such as synthetic inhibitors or natural bioactive compounds, offer diverse mechanisms of proteostasis modulation, even though considerable effort must be dedicated to evaluating the molecular basis of their mechanism of action and to elucidating their true applicability within clinical practice.

Citicoline (cytidine-5′-diphosphocholine), a natural intermediate in the synthesis of membrane phospholipids, is proposed as a neuroprotector and is widely used in glaucoma treatment, and showed multi-faceted cytoprotective properties against OS. Citicoline was shown to act as an allosteric modulator of proteasome activity in vitro and in cultivate cells, enhancing its capacity to eliminate ROS-derived oxidized proteins, thus contributing to restore cell homeostasis [112].

The promising anti-cataract agent Acetyl-L-carnitine prevents homocysteine-induced promoter demethylation in the human lens epithelial cell, which is a process that would otherwise induce Nrf2/Keap1-mediated proteasome activation [113]. The natural compound curcumin (1,7-bis-[4-hydroxy-3-methoxyphenyl]-1,6-heptadiene-3,5-dione) often proposed in biomedicine for its therapeutic potentiality is also a candidate agent for several eye diseases. A more bioavailable curcumin dispersed with polysaccharide nanoparticles (Theracurmin^®^ (Theravalues Corporation, Tokyo, Japan) or nano-curcumin) acts as a subtle proteasome modulator in RPE cells. In particular, the treatment with non-cytoxic doses induces the activity of immunoproteasome, with changes in the expression of proteasome-related subunits [114].

Even systemic anti-inflammatory agents, like a benzofuroxane derivative, which acts as an aldose reductase inhibitor, were found to ameliorate uveitis by decreasing the expression of ubiquitin and proteasome subunits, thereby mitigating the overall pro-inflammatory actions of the UPS [115].

Nature-inspired hybrids (NIH1-3) treatment, which induce Nrf2-mediated protection against OS, predisposes ARPE-19 cells to a better response to following exposure to proteasome and autophagy inhibitors, suggesting further investigation into their cytoprotective properties [116]. Finally, the wealth of small molecules and natural products known from cancer research represents a vast resource for new ocular treatments [117]. Among these, the anti-oxidant oleuropein from olives not only acts as a potent proteasome activator, accelerating the degradation of oxidized proteins [118,119]. The flavonol morin, which acts as ROS scavenger, restores the reduced chymotrypsin-like activity of proteasome following H_2_O_2_ exposure [120].

All these data collectively underscore the significant therapeutic potential of compounds that act by leveraging proteasome activity and its downstream pathways to effectively counteract OS, inflammation, and cellular dysfunction, as mentioned in a short summary shown in Table 1. This strategy points towards the development of novel pharmacological approaches for the management of eye diseases, particularly those involving a neurodegenerative etiology. However, it is important to recall that the biological consequences of proteasome modulation under OS remain incompletely resolved. In fact, in certain experimental settings, transient or mild UPS inhibition elicits adaptive, cytoprotective responses, largely mediated by autophagy induction and anti-oxidant signaling, whereas in others, more sustained or pronounced inhibition exacerbates proteotoxic stress and cellular vulnerability. These divergent outcomes likely depend on factors such as inhibitor dose, exposure time, cellular redox burden, and the capacity of autophagy to compensate reduced UPS activity. Clarifying these context-dependent effects is therefore essential for defining a true therapeutic window and for guiding the rational development of proteasome-targeting strategies in ocular disease.

## 6. Conclusions and Perspective

The convergence of OS and UPS dysfunction represents a crucial and tightly interconnected pathogenic axis underpinning the etiology of numerous disorders, including those affecting the ocular system. OS generates a proteotoxic burden that overwhelms UPS capacity, while UPS impairment further amplifies the accumulation of damaged or misfolded proteins, thereby exacerbating redox imbalance and perpetuating ROS production [121]. Although the central role of this reciprocal interaction in cellular homeostasis is well established, the intricate molecular details governing this bidirectional relationship remain a significant knowledge gap, particularly in ocular tissues with high metabolic demand such as the retina. Importantly, several unresolved mechanistic questions remain open, including the precise triggers governing the shift from adaptive to maladaptive proteasome responses under OS, the upstream redox-sensitive signaling nodes that regulate immunoproteasome induction, and the identity of E3 ligases whose activity is modulated by oxidative cues. A deeper elucidation of these key aspects is therefore essential to identify actionable therapeutic nodes. Moreover, current therapeutic approaches reveal intrinsic limitations. Broad-spectrum anti-oxidants, while conceptually appealing, often exhibit limited efficacy due to poor tissue selectivity, redundancy within endogenous anti-oxidant networks, insufficient bioavailability in ocular compartments, as the blood–retinal and blood–aqueous barriers restrict effective pharmacological concentrations within target tissues. These limitations are reflected in the variable and often modest outcomes observed in clinical trials of AMD, diabetic retinopathy, and glaucoma, where systemic anti-oxidant supplementation has shown inconsistent benefits and, in some cases, paradoxically, potential pro-oxidant effects at high doses [122]. Likewise, non-selective modulation of the proteasome has produced encouraging results: proteasome-activating compounds have demonstrated the capacity to enhance proteostasis balance and to attenuate age-related protein aggregation. Regardless, their clinical translation remains constrained by off-target or systemic adverse effects. In fact, non-specific activation of the proteasome may disrupt essential key cellular pathways, affecting cell-cycle control, inflammatory signaling, or synaptic function [6]. This aspect acquires particular relevance in the eye, where excessive proteasome stimulation could alter RPE viability, compromise photoreceptor support, or exacerbate inflammatory cascades. Moreover, systemic administration of proteasome modulators poses risks of systemic adverse events, such as hematological toxicity, peripheral neuropathy, and metabolic disturbances, underscoring the need for locally delivered compounds [6,7]. To note, beyond these strategies, increasing attention has been directed toward pharmacological activation of Nrf2, the master regulator of anti-oxidant and cytoprotective responses, as extensively reviewed elsewhere. Anyway, despite promising preclinical evidence in models of glaucoma, AMD, and diabetic retinopathy, chronic or generalized Nrf2 activation raises concerns related to metabolic reprogramming and potential interference with physiological proteasomal turnover [123]. With these considerations in mind, future research should focus on highly selective pharmacological agents designed not merely to scavenge ROS globally, but also to (i) selectively modulate proteasome activity to enhance degradation of oxidatively modified or aggregation-prone substrates, (ii) targeting immunoproteasome to offer the opportunity to modulate inflammatory and redox pathways with greater specificity than constitutive proteasomes; (ii) targeting redox-sensitive E3 ligases that represent a largely unexplored class of regulators with high potential for selective intervention; and (iv) fine-tuning Nrf2 signaling to restore redox equilibrium without inducing sustained supraphysiological activation. Additionally, the development of specific ocular delivery systems (e.g., intravitreal formulations, nanocarriers, sustained-release implants) may mitigate systemic toxicity and improve therapeutic outcomes. The identification of patient subgroups with well-defined redox or proteasomal dysfunction profiles could also enhance the clinical success of personalized interventions. Taken together, the complex and multilayered crosstalk between OS pathways and proteasome regulation and the actual limitations and gap discussed throughout this review provide a coherent framework for future translational efforts. A deeper understanding of how OS reshapes UPS function in specific ocular cell types should offer concrete entry points for developing targeted therapies capable of restoring proteostasis, mitigating inflammation, and ultimately slowing retinal degeneration.

## Figures and Tables

**Table 1:** Summary of ocular diseases, anti-oxidants and proteasome activity modulation.

Ocular Disease	OS and Proteasome Modulation	Experimental Evidence	Anti-Oxidant	Refs.
**Retinal-ischemia**	Saline solution infusion and retina blanching in animal model. Inhibition of proteasome activity and anti-oxidative effects.	Evaluation of functional changes in the retina by Electroretinogram, Western blot, mRNA expression, proteasome activity in retina.	Bortezomib	[105]
**Diabetic retinopathy**	Diabetic animal model and human cell lines incubated with high glucose. Retinal vascular injury after OS and proteasome inhibition.	Upregulation of Nrf2, and increased level NADPH-quinone oxidoreductase, and Heme Oxygenase.	MG-132	[107]
**Age-related macular degeneration**	Oxidative stress by H2O2 and proteasome inhibition-like effect.	Induction of autophagy, pro-survival and anti-inflammatory stimuli in ARPE-19 cells.	Resveratrol	[110]
	Epoximicin and IL-1 inducers.	Reduced cellular cytotoxicity and IL-1 levels in both proteasome inhibitors epoxomicin and MG-132 treated ARPE-19 cells.	N-acetyl-L-cysteine	[108,111]
	Proteasome inhibition and concomitant anti-oxidant use.			
	Compounds that are ROS inducers in ARPE-19 cells. Influence of proteasome subunit complexes in RPE cells.	Both nano-curcumin and curcumin exert concentration-dependent changes in the activity of proteasome individual subunits in RPE in vitro.	Curcumin	[114]
			nano-curcumin	
	NIH1–3 treatments predisposed ARPE-19 cells to a better response to following exposure to proteasome and autophagy inhibitors.	NIH compounds protect cellular viability of ARPE-19 upon prolonged proteasome and autophagy dysfunction.	Nature-inspired hybrids (NIH1-3)	[116]
	Cell line exposed to menadione or 4-hydroxynonenal. Inhibition of proteasome activity.	Toxicity induced by oxidative stressors and protective effect of protesome inhibitors. Activation of autophagy pathway. Inhibition of PI3K/Akt/mTOR pathway.	Clasto-lactcystin-lactone	[106,109]
**Damaged retinal ganglion cells and glaucoma**	Allosteric modulation of proteasome activity	Influence on proteolytic activity of the 20S proteasome on synthetic and natural substrates, functioning as a bimodal allosteric modulator.	Citicoline	[112]
**Cataract**	Effects of homocysteine in HLECs. Demethylation of Keap1 promoter DNA and increasing Nrf2 activity for proteasomal degradation.	Prevent demethylation in DNA promoter region induced by homocysteine in human lens epithelial cells.	Acetyl-L-carnitine	[113]
**Uveitis**	Rats treated for LPS induced uveitis. Decreased the expression of the ubiquitin, 20S and 26S proteasome subunits in uveitic eyes.	Effects of the aldose reductase inhibitor benzofuroxane derivative on the biochemical and tissue alterations induced by endotoxic uveitis in rats.	Benzofuroxane derivative	[115]
**Dry eye syndrome**	Hyperosmotic and OS on rabbit corneal epithelial cells. Proteasome activator.	Oleuropein was able to control the effects of hyperosmolarity on ocular surface cells and to prevent OS-induced loss of cell viability.	Oleuropein	[118]

## Abbreviations

AGEs	Advanced Glycation End Products;
AKT	Protein Kinase B;
ALP	Autophagy–Lysosomal Pathway;
AMD	Age-Related Macular Degeneration;
Ang II	Angiotensin II;
APDC	(2R,4R)-4-Aminopyrrolidine-2,4-dicarboxylic acid;
AT1R	Angiotensin II Type 1 Receptor;
ATP	Adenosine Triphosphate;
ATRAP	Angiotensin II Type 1 Receptor–Associated Protein;
C-L	Caspase-like;
Cbx4	Chromobox Homolog 4;
ChT-L	Chymotrypsin-like;
DR	Diabetic Retinopathy;
dKO	Double Knockout;
E1	Ubiquitin Ligase Enzyme E1;
E2	Ubiquitin Ligase Enzyme E2;
E3	Ubiquitin Ligase Enzyme E3;
EI24	Etoposide-induced protein 2.4 homolog;
ER	Endoplasmic Reticulum;
GSTP1	Glutathione S-Transferase P1;
H_2_O_2_	Hydrogen Peroxide;
HIF-1α	Hypoxia-Inducible Factor 1-alpha;
HSP70	Heat shock protein 70;
IFN-γ	Interferon gamma;
IkBα	Nuclear factor of kappa light polypeptide gene enhancer in B-cells inhibitor alpha;
IKK	IκB Kinase;
IL	Interleukin;
IOP	Intraocular Pressure;
Keap1	Kelch-like ECH-associated Protein 1;
LC3	Microtubule-Associated Protein 1A/1B-Light Chain 3;
LMP	Low Molecular Weight Protein;
MAPK	Mitogen-activated protein kinase;
MAPT	Microtubule-associated protein tau;
MCP-1	Monocyte Chemoattractant Protein-1;
mTORC1	Mammalian Target of Rapamycin Complex 1;
NAC	N-Acetyl-L-Cysteine;
NF-κB	Nuclear Factor kappa-light-chain-enhancer of Activated B-Cells;
NIH	Nature-inspired hydrids;
NLRP3	NOD-, LRR- and Pyrin Domain-Containing Protein 3;
NQO1	NAD(P)H Quinone Dehydrogenase 1;
Nrf2	Nuclear Factor Erythroid 2–Related Factor 2;
OS	Oxidative Stress;
PA28	Proteasome Activator 28;
PA700	19S Proteasome Regulatory Complex;
PAF	Platelet-Activating Factor;
PGC-1α	Peroxisome Proliferator-Activated Receptor Gamma Coactivator 1-alpha;
PGPH	Peptidylglutamyl-peptide hydrolyzing;
PI3k	Phosphoinositide 3-kinase;
PIASy	Protein Inhibitor of Activated STAT Y;
PIPs	Protesome interacting proteins;
PKC	Protein Kinase C;
PPARα	Peroxisome Proliferator-Activated Receptor Alpha;
PSMB5	Proteasome catalytic β5 subunit;
PTEN	Phosphatidylinositol-3,4,5-trisphosphate 3-phosphatase;
REDD1	Regulated in development and DNA damage response 1;
RGC	Retinal Ganglion Cell;
RNS	Reactive Nitrogen Species;
RNF114	RING Finger Protein 114;
ROS	Reactive Oxygen Species;
RPE	Retinal Pigment Epithelium;
SQSTM1/p62	Sequestosome 1;
SUMO	Small Ubiquitin-like Modifier;
tBHP	tert-butyl hydroperoxide;
TM	Trabecular Meshwork;
T-L	Trypsin-like;
UBE3D	Ubiquitin protein ligase E3D;
UPS	Ubiquitin–Proteasome System;
